# Effects of proarrhythmic drugs on relaxation time and beating pattern in rat engineered heart tissue

**DOI:** 10.1007/s00395-014-0436-7

**Published:** 2014-09-11

**Authors:** Alexandra Eder, Arne Hansen, June Uebeler, Thomas Schulze, Christiane Neuber, Sebastian Schaaf, Lei Yuan, Torsten Christ, Marc A. Vos, Thomas Eschenhagen

**Affiliations:** 1 Department of Experimental Pharmacology and Toxicology, University Medical Centre Hamburg-Eppendorf, Martinistraße 52, 20246 Hamburg, Germany; 2 DZHK (German Centre for Cardiovascular Research), Partner site Hamburg/Kiel/Lübeck, Martinistrasse 52, 20246 Hamburg, Germany; 3 Danish National Research Foundation Centre for Cardiac Arrhythmia, Department of Biomedical Sciences, University of Copenhagen, Copenhagen, Denmark; 4 Department of Medical Physiology, UMC Utrecht, Yalelaan 50, 3584 CM Utrecht, The Netherlands

**Keywords:** Arrhythmia, Torsades des pointes, Drugs, In vitro screening, Engineered heart tissue

## Abstract

The assessment of proarrhythmic risks of drugs remains challenging. To evaluate the suitability of rat engineered heart tissue (EHT) for detecting proarrhythmic effects. We monitored drug effects on spontaneous contractile activity and, in selected cases, on action potentials (sharp microelectrode) and Ca^2+^ transients (Fura-2) and contraction under electrical pacing. The I_to_-blocker inhibitor 4-aminopyridine increased action potential duration and T2 and caused aftercontractions, which were abolished by inhibitors of ryanodine receptors (RyR2; JTV-519) or sodium calcium exchanger (NCX; SEA0400). 77 Drugs were then tested at 1-10-100× free therapeutic plasma concentrations (FTPC): Inhibitors of I_Kr_, I_Ks_, I_to_, antiarrhythmics (8), drugs withdrawn from market for torsades des pointes arrhythmias (TdP, 5), drugs with measurable (7) or isolated TdP incidence (13), drugs considered safe (14), 28 new chemical entities (NCE). Inhibitors of I_Kr_ or I_Ks_ had no effect alone, but substantially prolonged relaxation time (T2) when combined at high concentration. 15/33 drugs associated with TdP and 6/14 drugs considered non-torsadogenic (cibenzoline, diltiazem, ebastine, ketoconazole, moxifloxacin, and phenytoin) induced concentration-dependent T2 prolongations (10-100× FTPC). Bepridil, desipramine, imipramine, thioridazine, and erythromycin induced irregular beating. Three NCE prolonged T2, one reduced force. Drugs inhibiting repolarization prolong relaxation in rat EHTs and cause aftercontractions involving RyR2 and NCX. Insensitivity to I_Kr_ inhibitors makes rat EHTs unsuitable as general proarrhythmia screen, but favors detection of effects on I_to_, I_Ks_ + I_to_ or I_Ks_ + I_Kr_. Screening a large panel of drugs suggests that effects on these currents, in addition to I_Kr_, are more common than anticipated.

**Electronic supplementary material:**

The online version of this article (doi:10.1007/s00395-014-0436-7) contains supplementary material, which is available to authorized users.

## Introduction

Proarrhythmic side effects of drugs can be life-threatening and have led to a number of drug withdrawals from the market [17]. Predicting the arrhythmogenic potential in preclinical drug development is difficult for several reasons [25, 37]. (1) Mechanisms of arrhythmias are complex. This is exemplified by the fact that not only loss-of-function, but also gain-of-function mutations of K^+^- and Na^+^-channels can cause arrhythmias by promoting ectopic activity, dispersion, and/or re-entry circuits [1]. This corresponds with clinical experiences that both class I (Na^+^-channel blockers) and class III (K^+^-channel blockers) antiarrhythmic drugs have significant proarrhythmic effects [10]. In addition, dysfunction of the sarcoplasmic reticulum Ca^2+^-release channel (RyR2) or the SR Ca^2+^-storage protein calsequestrin underlie catecholaminergic polymorphic ventricular tachycardia, characterized by increased ectopic automaticity in situations of stress. (2) The heart is equipped with several safety mechanisms, explaining why one hit is rarely sufficient to cause symptomatic arrhythmias. Even patients with inherited rhythm disorders experience clinically relevant arrhythmias relatively late in life and/or only under certain trigger situations such as increased sympathetic drive, hypokalemia, drugs, ischemia, or myocardial scars. (3) The existing preclinical test systems have shortcomings [14, 25, 37]. In current routine, new chemical entities (NCE) are tested on cells overexpressing the human eag-related gene (hERG), and those with significant inhibitory activity are excluded from further development. Whereas hERG-tests have documented high sensitivity and specificity for this single ion current and the principal relevance for hERG-inhibition in causing torsades des pointes (TdP) is undisputed, the predictive value of this test is limited [21, 31]. Several drugs have hERG-inhibitory activity without being associated with TdP-arrhythmias (e.g., verapamil). Others have no relevant hERG-activity at clinically used concentrations, but increase the risk of arrhythmias (e.g., mefloquine and phenytoin; [31]).

The Food and Drug Administration and the European Medicines Agency currently recommend an integrated risk assessment, which includes the results of several experimental tests (e.g., hERG, rabbit Purkinje fibers, and dog telemetry) as well as in silico and clinical data [11, 12, 38]. The content of current tests may be improved by characterizing NCEs in a whole panel of cell lines, each expressing a different cloned ion channel. This approach provides a more comprehensive picture of a drugs channel-affecting activity [3, 42], but needs modeling to predict the integrated effect. An alternative strategy is to test drugs directly on cardiac myocytes or more complex cardiac tissues as the “real substrate” for arrhythmias, assuming that any reproducible effect is relevant, independent of its exact mechanism. Unfortunately, isolated adult cardiac myocytes do not beat and, similar to Purkinje fibers or Langendorff-perfused hearts, cannot be examined in large series. Instrumented dogs or rabbits are the most valid models, but cannot be used for screening large number of NCEs, both for ethical and financial reasons.

We have recently developed an automated miniaturized drug screening assay based on our EHT technology and neonatal rat cardiac myocytes, which appears to combine some of the advantages of a relatively intact 3D cardiac tissue, availability at large numbers, robustness and high-content readout, particularly analysis of contractile force and kinetics [7, 16, 20]. Experiments with a limited number of model compounds indicated assay sensitivity to detect proarrhythmic effects of drugs [16]. The aim of the present study was to systematically determine the predictive value of the assay by testing a larger number of clinically used compounds with characterized hERG-inhibitory activity and proarrhythmic potential in humans [31] and randomly picked NCEs as well as underlying mechanisms.

## Materials and methods

The investigation conforms to the guide for the care and use of laboratory animals published by the NIH (Publication No. 85-23, revised 1985). A detailed description of methods can be found in the supplemental file.

### Cell isolation and EHT-generation

Heart cells of postnatal d0–d3 Wistar rats and EHTs were prepared as previously described [16, 44]. Fresh neonatal heart cells were mixed with medium, fibrinogen and thrombin, and casted into strip-format (12 × 3 × 3 mm) molds in agarose, in which pairs of elastic silicone posts were placed from above. EHTs maintained for up to 4 weeks.

### Measurement of contractile parameters

Contractile parameters were evaluated as previously described [16]. In principle, the 24-well-plates with EHTs (14–21 days) were put in a gas-, temperature-, and humidity-controlled incubator with glass roof and customized software-controlled video camera placed on top. Contractile parameters of spontaneously beating EHTs were evaluated using an automated figure recognition algorithm. Deflection of the silicone posts was recorded over time and, based on post geometry and elastic modulus of the silicone, used to calculate force, frequency, fractional shortening, contraction and relaxation time (bpm, T1 [from 20 % to peak] and T2 [from peak to 20 %], respectively).

### Drug screening—video optical analysis

**Fig. 1:**
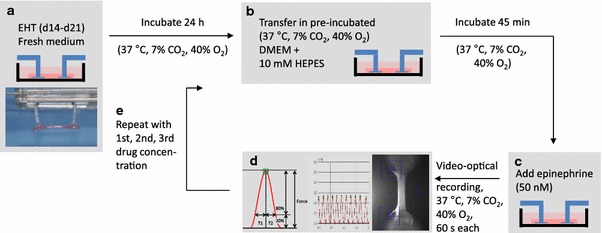
Schematic illustration of the standard operation procedure for evaluating drug effects. EHTs were subjected to measurements at day 14–21. One day before measurement culture medium was changed. Before evaluation, EHTs were transferred to fresh, preincubated (37 °C, 7 % CO_2_, 40 % O_2_), serum-free DMEM supplemented with 10 mM HEPES for pH steadiness, and incubated for 45 min. Epinephrine (50 nM) was added and contractile parameters were analyzed by the video optical system. Thereafter, EHTs were transferred to fresh preincubated DMEM, including HEPES plus the first concentration of a drug, and incubated for 45 min. Epinephrine was added, measurements were done and this circuit started again with the second concentration of the drug

All measurements were performed with 14–21 day old EHTs in fresh serum-free DMEM (Biochrom F04115), supplemented with 10 mM HEPES for pH-steadiness, preincubated at 37 °C, 40 % O_2_, 7 % CO_2_, 90 % humidity for 2 h. Measurements were done routinely 1 day after feeding with standard EHT medium. The drugs were analyzed in three different concentrations (45 min each, cumulative, 1–100× free therapeutic plasma concentration [FTPC]). For drug details including solvents see Supplement Table 1. Prior to each measurement, 50 nM epinephrine (Sigma E4643) was added to each well to simulate “physiological” conditions and enhance the likelihood of contractile activity within the 60 s recording time. Drugs were added under sterile conditions. After 45 min incubation in a standard incubator, EHTs were transferred to the video optical system (Fig. 1). Due to a sequential mode of measurement (total time ~30 min), incubation time varied from the first to the last EHT (45–75 min).

### Measurements under perfusion and electrical stimulation, Ca^2+^-transients, action potentials

Intracellular Ca^2+^-transients were analyzed in parallel with force under electrical stimulation and continuous perfusion using a novel setup as described previously [35]. The setup consisted of an inverted microscope, a temperature- and O_2_/CO_2_-controlled chamber for the 24-well EHT plate, a flow rate-controlled perfusion system, platinum–iridium wire electrodes for field stimulation, a fluorescence light source (IonOptix Hyperswitch), a photomultiplier, video cameras and software (both IonOptix) for the evaluation of contractile activity (edge detection mode). Experiments were done at 4 ml flow/min (per well) and 2–4 Hz stimulation in modified Tyrode’s solution.

For Fura loading, EHTs were incubated in Tyrode’s solution containing 10 µM Fura2-AM (Invitrogen F1221) and Cremophor EL (0.75 %; Sigma C5135) for 2 h at 37 °C. The ratio of light emission (510 nm) at excitation with 340 and 380 nm light (F340/380 ratio) was used as an index of cytosolic Ca^2+^-concentration.

Action potentials (APs) were recorded with standard intracellular microelectrodes in intact EHTs. Bath solution contained (in mM): NaCl 127, KCl 4.5, MgCl_2_ 1.5, CaCl_2_ 1.8, glucose 10, NaHCO_3_ 22, NaHPO_4_ 0.42, equilibrated with O_2_-CO_2_ [95:5] at 36.5 ± 0.5 °C, pH 7.4. Preparations were field-stimulated for at least 1 h (2 Hz) before data acquisition. APs were analyzed off-line using the LabChart^®^ software (ADInstruments, Spechbach, Germany).

### Statistical analysis

Data were expressed as mean ± SEM. Statistical differences were analyzed using the one-way analysis of variance (ANOVA) followed by the Dunett’s (all compared to baseline) or Tukey’s (all compared to all) adjustment for post hoc multiple comparison, or by paired or unpaired Student’s *t* test, as indicated in the legend of each figure. Results were considered statistically significant if a paired Student’s *t* test revealed a *p* value of less than 0.05 and the deviation from baseline was at least 15 %. This limit was defined after initial series of experiments had shown that formally significant (*t* test), but not concentration-dependent effects of drugs often amounted to ±11 %. Further support for the 15 % threshold came from quantifying the mean ± SD of all baseline measurements (*n* = 221 independent EHTs), which amounted to 99.6 ± 11.4 % (SEM ± 0.77 %).

## Results

### Mechanisms of twitch prolongation and irregular beating pattern

**Fig. 2:**
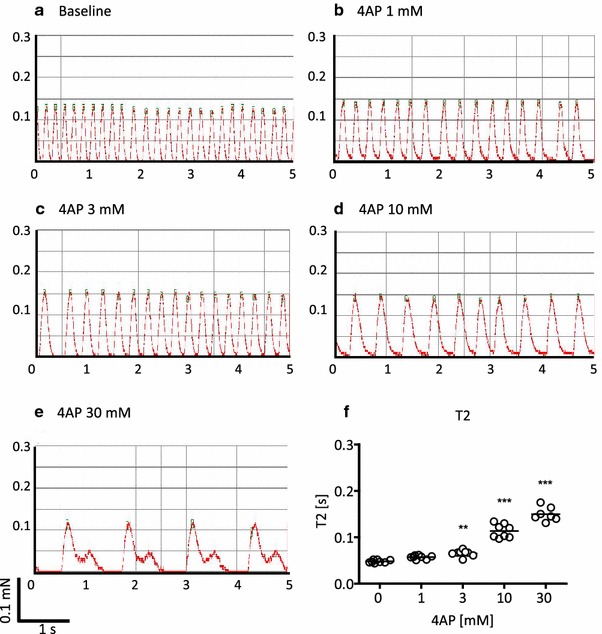
Concentration-dependent effects of the I_to_-blocker 4-aminopyridine (4AP). **a**–**e** Representative original recordings of spontaneously beating EHTs in the absence (baseline, only epinephrine 50 nM) and presence of increasing concentrations of 4AP. **f** statistical evaluation of relaxation time (T2; each dot represents one analyzed EHT; **p < 0.003, ***p < 0.0001 vs. baseline one-way ANOVA+ Dunnett’s post test). The concentration of 4AP was cumulatively increased with 45 min incubation steps per concentration. Note the concentration-dependent prolongation of contraction twitches and the aftercontractions at high concentrations

**Fig. 3:**
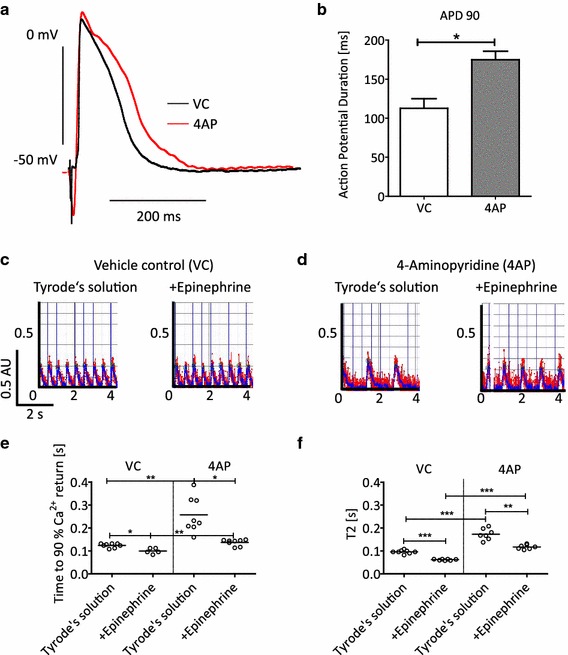
Effect of 4-aminopyridine (4AP; 10 mM), epinephrine (50 nM) or their combination on action potential duration (APD_90_), Fura-2 F340/380 ratio and relaxation time (T2) of electrically stimulated EHTs (2 Hz). For APD_90_ measurements two independent groups of EHTs (*n* = 3, each) were directly perfused with vehicle control (VC) or 4-aminopyridine (4AP), electrically stimulated and contractions recorded. Preparations were stimulated for at least 1 h before drug exposure and data acquisition. For Fura-2 F340/380 ratio and T2 the two independent groups (*n* = 8, each) were preincubated with VC or 4AP. Thereafter, EHTs were perfused and electrically stimulated (2 Hz; baseline) for 10 min, before epinephrine (50 nM) was added to the perfusion system. **a** Average peaks for action potentials in the absence (VC, *black line*) and presence of 4AP (*red line*). **b** Statistical evaluation of APD_90_. **c** Original recordings of F340/380 ratio transients in the absence (VC, *left*) and presence of epinephrine (*right*). **d** Original recordings in the presence of 4AP alone (*left*) and 4AP plus epinephrine (*right*). The *red line* displays the unfiltered signal, whereas the *blue line* displays the filtered signal. **e** Statistical evaluation of time to 90 % return of the F340/380 ratio and F T2. Each point represents one EHT. *p < 0.05, **p < 0.003, ***p < 0.0001, Student’s *t* test, paired for comparison within one group (baseline vs. epinephrine) and unpaired for comparison between the two groups (VC vs. 4AP); y-axes for C, D: F340/380 ratio in arbitrary units (AU). Note that 4AP increased T2 and time to 90 % return of the F340/380 ratio, both in the absence and presence of epinephrine. Epinephrine alone shortened T2 and F340/380 transients. Note also the failure of capture under 4AP

In a previous study with EHTs, we found that the experimental I_Ks_-inhibitor chromanol 293b and two drugs known to inhibit I_Kr_ and cause TdP in humans, quinidine and erythromycin, caused concentration-dependent prolongations of relaxation time T2 [16]. These observations suggested that T2 is a useful surrogate for drug-induced prolongations of repolarization and proarrhythmic effects. To test this hypothesis, we evaluated the effects of the I_to_-inhibitor 4-aminopyridine (4AP; IC_50_ on I_to_ in adult rat ventricular myocytes: 980 µM [41]) on EHTs. 4AP prolonged T2 concentration dependently reaching significance at 3 mM (Fig. 2). At 30 mM, EHTs showed extremely prolonged relaxation (+502 %), comparable to what have been seen previously with chromanol (+710 %; [16]). Prolonged contractions can either be caused by altered intracellular Ca^2+^ transients or myofilament response to Ca^2+^. To differentiate between these mechanisms, EHTs were subjected to sharp electrode measurements of action potentials and calcium transients (Fura-2; Fig. 3). Action potential characteristics under basal conditions and electrical stimulation were similar as described previously in this model [16]. 4AP increased action potential duration (APD_90_) from a mean of 113 to 175 ms (Fig. 3a, b). 4AP also prolonged intracellular Ca^2+^ transients, whereas the β-adrenergic agonist epinephrine shortened it, both in the absence and presence of 4AP (Fig. 3c–f). Quinidine, a still prescribed drug for the treatment of atrial fibrillation, also increased APD_90_ from 179 to 232 ms at 100 µM (Supplemental Fig. 5d). These data supported the interpretation that alterations in T2 reflect similar changes in APD and the kinetics of intracellular Ca^2+^ transients. Accordingly, a high concentration of caffeine, known to open ryanodine receptors (RyR2) in the sarcoplasmic reticulum (SR), reduced beating rate and induced a large and wide twitch (Supplemental Fig. 1).

**Fig. 4:**
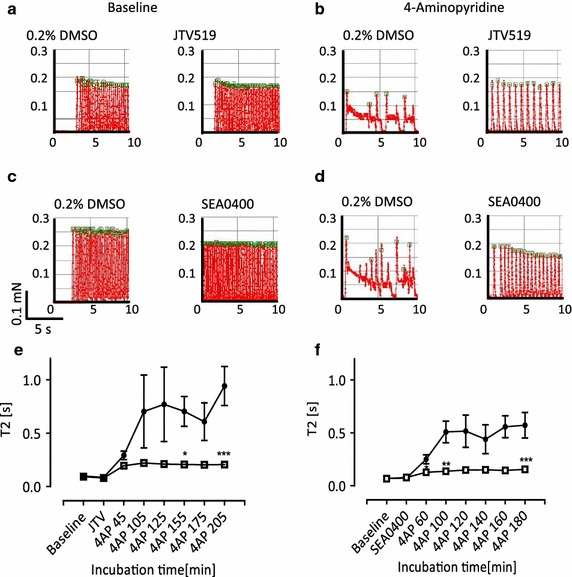
Effect of inhibitors of the ryanodine-receptor (JTV519 2 µM) or the sodium calcium exchanger (NCX, SEA0400 2 µM) on 4-aminopyridine (4AP 10 mM)-induced prolongations of relaxation and aftercontractions in spontaneously beating EHTs. **a**–**d** Original recordings of the contractile activity of EHT in the absence of drugs (**a**, **c** vehicle control), JTV519 (**a**, JTV519), SEA0400 (**c**, SEA0400), 4AP alone (**b**, **d** vehicle control) or in the presence of JTV519 (**b**, JTV5019) or SEA0400 (**d**, SEA0400). **e**, **f** Time course of the effect of 4AP in the absence (*black dots*) or presence of JTV519 (**e** *clear squares*) or SEA0400 (**f** *clear squares*). *n* = 8; data are expressed as mean ± SEM; *p < 0.05, **p < 0.003, ***p < 0.0001 vs. vehicle control, one-way ANOVA+ Tukey’s post test. Note that both inhibitors prevented the marked 4AP-induced T2 prolongation and after contractions, but did not completely normalize T2 or beating frequency

4AP not only prolonged T2, but also induced beat-to-beat irregularities, variations of twitch amplitude and aftercontractions falling into the relaxation phase of prolonged twitches (Fig. 4). To investigate the role of different cellular effector systems in the T2-prolonging effect of repolarization-prolonging compounds, we determined the effect of 4AP in the absence and presence of tetrodotoxin (I_Na_), tetracaine (I_Na_), verapamil (I_Ca_), thapsigargin (SERCA), SEA0400 (sodium calcium exchanger, NCX) or JTV519 (RyR2), respectively. Verapamil had no discernible effect, thapsigargin, tetrodotoxin and tetracaine all increased the T2-prolonging effect of 4AP (Supplemental Fig. 2). In contrast, SEA0400 and JTV519 reduced the effect of 4AP (Fig. 4). Both compounds completely abolished beating irregularity and aftercontractions, but only partially T2-prolongation. T2 values were 127 (SEA + 4AP) vs. 78 ms (SEA alone) and 195 (JTV + 4AP) vs. 80 ms (JTV alone). This suggests that part of the 4AP-induced T2-prolongation was a direct consequence of action potential prolongation, whereas the SEA- and JTV-sensitive after contractions and pronounced T2-prolongations were mediated by RyR2 and NCX. Similar data were obtained with thioridazine (30 µM, data not shown).

### Role of I_Kr_ and I_Ks_ in T2 prolongations

**Fig. 5:**
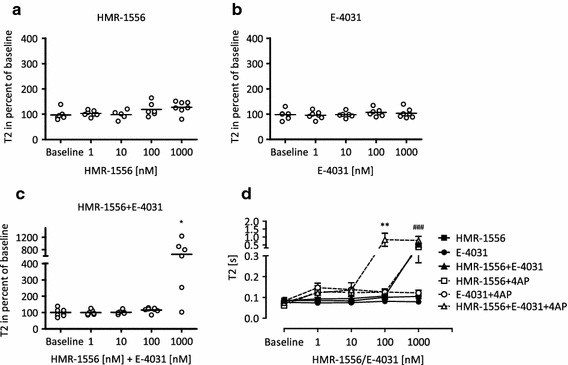
Role of I_Ks_ and I_Kr_ in rat EHTs. Statistical evaluation of relaxation time (T2) in the presence of HMR-1556 (**a**), E-4031(**b**) or the combination of both (**c**). Neither HMR-1556 nor E-4031 alone affected T2, but the combination of both led to a marked increase of T2 at the highest concentration (each 1 µM). **d** shows the concentration response curves of HMR-1556, E-4031, and the combination of both in the absence (*black lines*) and presence of 4AP (*dashed lines*; 3 mM). In the presence of 4AP, the concentration response curves for HMR-1556 and HMR-1556 + E-4031 were shifted to the left, whereas the curve for E-4031 alone was not affected. **a**–**c** each dot stands for one analyzed EHT; *p < 0.05 paired Student’s *t* test vs. baseline (50 nM epinephrine); **d** *n* = 4–8, data are expressed as mean ± SEM; **p < 0.003, ***p < 0.0001 vs. intervention + 4AP (^#^HMR-1556; *HMR-1556 + E-4031), one-way ANOVA + Tukey’s post test. Note that differences due to the presence of 4AP were not included

The role of I_Kr_ and I_Ks_ for action potential repolarization in rat heart is still poorly understood [32]. We studied the involvement of these two currents in rat EHTs by applying the reference I_Kr_ blocker E-4031 (IC_50_ 7.7 nM [43]) and the selective I_Ks_ blocker HMR-1556 (IC_50_ 10.5 nM [36]) alone or in combination (1–1,000 nM, Fig. 5). Neither E-4031 nor HMR-1556 affected T2 even at high concentrations (1,000 nM). The combined application also did not affect T2 at up to 100 nM, but caused a substantial increase at 1,000 nM (>8-fold). Given the high selectivity of E-4031 and HMR-1556 for I_Kr_ and I_Ks_ (HMR-1556 IC_50_ on I_to_: 33.9 µM; on I_Ca_: 27.5 µM; on I_Kr_: 12.6 µM [36]), respectively, the effect of the combination suggests a role of these two currents for determining the repolarization reserve in rat EHTs.

### Screening of proarrhythmic compounds under spontaneous beating

**Fig. 6:**
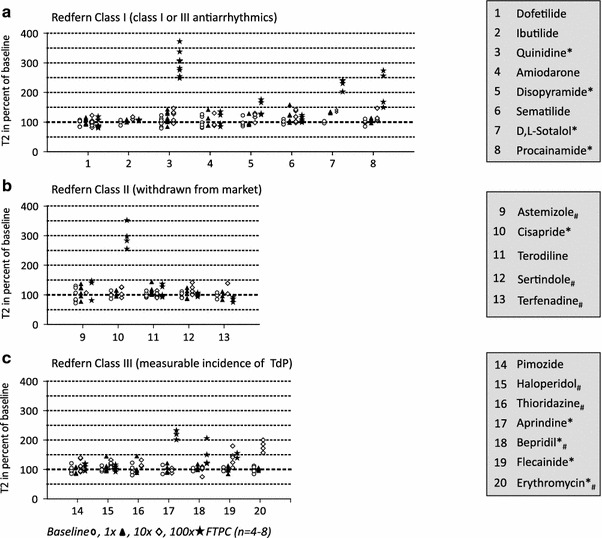
Effects of class I–III (Redfern) drugs on rat EHT relaxation time (T2). **a** Shows the effect of antiarrhythmics (class I), **b** of drugs which were withdrawn from the market (class II) and **c** of drugs with measureable incidence of TdP. Each symbol reflects one analyzed EHT. Different concentrations are indicated by different symbols (baseline , 1×, 10×, 100× FTPC). Statistically significant changes in T2 are indicated by an *asterisk* behind the drug name. Effects other than T2 prolongations are indicated by *hashtag* (e.g., thioridazine: irregular beating). Data are expressed as percent of baseline (50 nM epinephrine). *p < 0.05 paired Student’s *t* test vs. baseline and at least 15 % difference between means

**Fig. 7:**
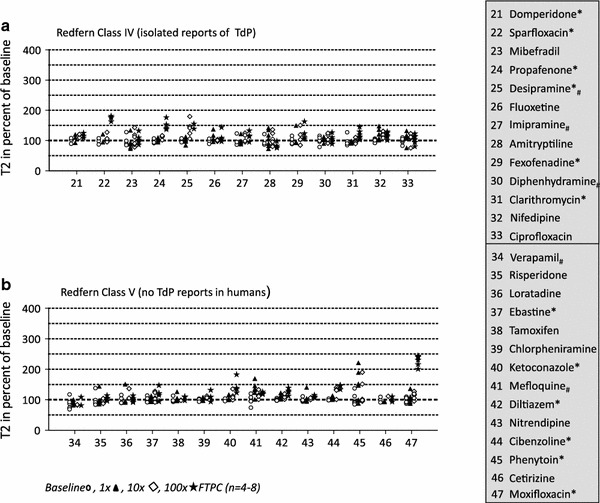
Effects of class IV–V (Redfern) drugs on rat EHT relaxation time (T2). **a** Shows the effect of drugs with isolated reports of TdP (class IV) and **b** with no reports of TdP in humans (class V). Each symbol reflects one analyzed EHT. Different concentrations are indicated by different symbols (baseline , 1×, 10×, 100× FTPC). Statistical significant changes in T2 are indicated by an *asterisk* behind the drug name. Effects other than T2 prolongations are indicated by *hashtag* (e.g., verapamil: negative inotropic effect). Data are expressed as percent of baseline (50 nM epinephrine). *p < 0.05 paired Student’s *t* test vs. baseline and at least 15 % difference between means

To test the usefulness of our screening system for the detection of proarrhythmic compounds, we analyzed a large panel of drugs associated with arrhythmias. The selection was made according to a list of drugs published by Redfern and colleagues [31] which related various levels of proarrhythmic risk with inhibition of I_Kr_ (hERG). We tested the effect of the 46 compounds of this list which were commercially available (details Supplemental Table 1) at 1-, 10-, and 100-fold FTPC, *n* = 4–8 each. In addition, we tested moxifloxacin, an important inhibitor of bacterial gyrases, associated with prolongation of the QT-interval, but not with arrhythmias [28]. The selection of compounds encompassed 8 clinically used antiarrhythmic drugs (Group I), 5 drugs withdrawn from the market for TdP (Group II), 7 drugs with measurable incidence of TdP in humans (Group III), 13 drugs with isolated reports of TdP (Group IV) and 14 drugs devoid of TdP reports and therefore considered safe (Group V; Figs. 6, 7). Under the experimental conditions (which included a ~EC_50_ [6] epinephrine concentration, 50 nM) and in the absence of interventional drugs, EHTs showed a typical and reproducible beating pattern, consisting of periods with high frequency (4.4–4.6 Hz, called “bursts”, 8–10 s length) and periods of contractile inactivity (30–40 s; Supplemental Fig. 3). Single twitch kinetics showed a mean ± SD (SEM) T1 and T2 of 67 ± 8 (±1) and 92 ± 18 (±2) ms, respectively. Given that the bursts occurred by chance either fully inside the 60 s recording window or only partially, the total number of beats per 60 s was relatively meaningless. In contrast, the frequency of beating in the burst was stable and systematically affected by epinephrine (+15–20 %) and carbachol (reversed epinephrine effect and induced partial stop; Supplemental Fig. 3).

Given our previous results with erythromycin and quinidine [16], we focused on T1, T2, and the beating pattern as possible surrogates for arrhythmias. A threshold of 15 % T2 prolongation was considered significant (see Statistics for details). Supplemental Fig. 4 depicts typical examples of drug responses, Figs. 6 and 7 show the T2 effect of 47 drugs according to the Redfern classification. Significant T2 prolongations were observed with 4/8 Redfern Class I drugs (antiarrhythmics; quinidine, disopyramide, sotalol, procainamide; Fig. 6a), 1/5 drugs withdrawn from the market due to TdP (Redfern Class II; cisapride; Fig. 6b), 4/7 drugs with measurable incidence of TdP (Redfern Class III; aprindine, bepridil, flecainide, erythromycin; Fig. 6c), 6/13 drugs with isolated TdP reports (Redfern Class IV; domperidone, sparfloxacin, propafenone, desipramine, fexofenadine, clarithromycin; Fig. 7a), and 6/14 drugs considered safe (ebastine, ketoconazole, diltiazem, cibenzoline, phenytoin, moxifloxacin; Fig. 7b). Whereas most drugs prolonged T2 only at 100× FTPC, phenytoin exerted this effect already at 1× FTPC. In addition, five compounds (bepridil, desipramine, imipramine, thioridazine, and erythromycin) induced irregular beating as the primary effect, characterized by a “sinusoidal” fluctuation of twitch amplitudes (Supplemental Fig. 4a). The effect was time dependent and was followed by complete stop of beating. It occurred at concentrations of 30–100 fold FTPC, i.e., 3–10 (−600 in case of thioridazine) fold hERG IC_50_. Bepridil, desipramine, and erythromycin increased T2 in addition to inducing beating irregularity.

**Fig. 8:**
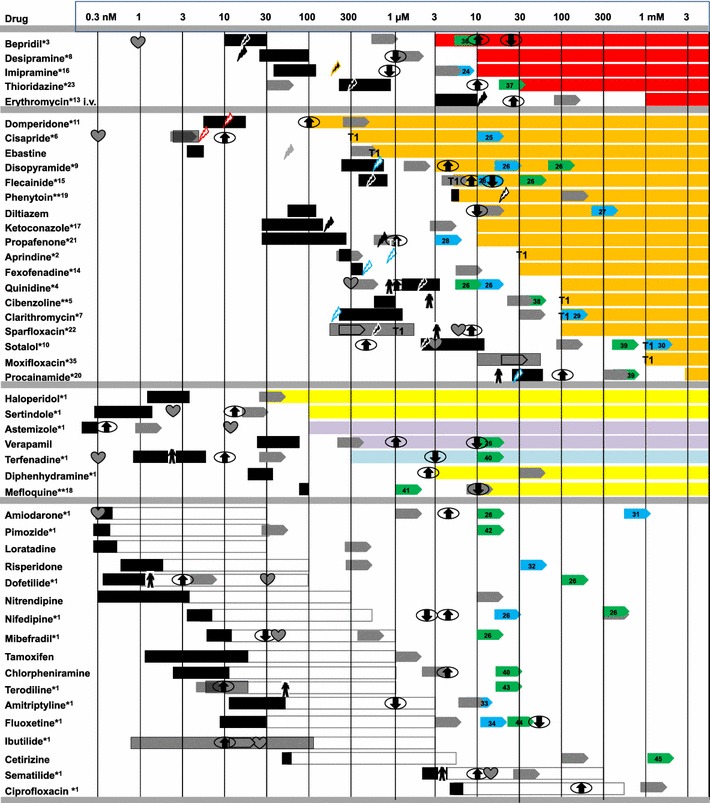
Grouping of drugs according to the type and the concentration-dependence of the effects on EHTs. All drugs were tested in 1×, 10×, and 100× free therapeutic plasma concentration (FTPC) in the presence of 50 nM epinephrine. The absolute concentration range is indicated in the first row. Group 1 encompasses drugs (*red*) that induced polymorphic arrhythmias, group 2 (*orange*) prolongations of relaxation (T2), group 3 prolongations of contraction (T1, *yellow*), shortening of T2 (*blue*), or negative inotropic effects (*violet*). Group 4 (*white*) drugs exerted no significant effect. The *colored bars* in group 1–3 range from the lowest tested concentration in which the indicated effect occurred (generally 10–100× FTPC) to 3 mM, regardless of whether this concentration has been tested. The *white bars* in group 4 indicate the tested concentration range. Also, indicated are hERG/I_Kr_ lowest published IC_50_ upward ; lowest published IC_50_ values on I_to_ upward ; lowest published IC_50_ values on I_Ks_ upward ; concentrations associated with 10–20 % increases in APD_90_ _;_ with decreases in APD_90_ _;_ QTc-prolongation in vivo ; increased QTc in humans; estimated plasma concentration (ep) for TdP case reports in humans ; ep for TdP case reports in combination with other drugs/diseases ; ep for TdP case reports in combination with a cytochrom P450 inhibitor ; ep for TdP case reports in children ; ep QTc case reports ; *asterisk* TdP in humans known; *double asterisk* other types of arrhythmias in humans known. The type of depiction and most information independent of EHTs were adopted from Redfern et al. (2003). Case reports (indicated by *asterisk*), estimated plasma concentration and IC_50_ values on I_to_/I_Ks_ were obtained by a literature search

In aggregate, the rat EHT responses categorized the 47 drugs in 4 groups (Fig. 8). Group 1 caused irregular beating as the main effect (*n* = 5), group 2 induced a concentration-dependent increase in T2 (*n* = 18). T2-effective concentrations were 5 fold (domperidone, disopyramide) to 100 fold FTPC (e.g., ebastine, diltiazem, sotalol, or moxifloxacin). Group 3 (*n* = 7) had variable effects on EHT contraction including prolongation of contraction time T1 (haloperidol, sertindole, diphenhydramine, and mefloquine), shortening of T2 (terfenadine) and a decrease in force (verapamil and astemizole). The negative inotropic effect of verapamil was already seen at 300 nM, approximately 4-fold FTPC. Group 4 encompassed 17 compounds without any effect on the parameters studied. It contained specific I_Kr_-blockers such as ibutilide and dofetilide, which was in contrast to the results obtained with human EHTs [33].

### Effects of drugs under electrical pacing

Drugs which caused prolongations of T2 also reduced beating rate in the bursts in many cases (Supplemental Fig. 4), raising the question which effect is the cause and which the consequence. We therefore measured EHT contractility under continuous electrical stimulation and perfusion and carefully evaluated the time course of the effects of quinidine (100 µM) and erythromycin (1 mM; Supplemental Fig. 5). Quinidine first induced a marked, time-dependent prolongation of T2 and a reduction in force, which was then followed by the development of a slower rhythm independent of pacing. Erythromycin also first prolonged T2, reduced force and then induced an autonomous chaotic contraction pattern. Effects of both drugs were fully reversible after washout. The data suggest that the drugs exert primary effects on processes underlying relaxation and that slowing of rate is a consequence.

### Screening of new chemical entities (NCEs) under spontaneous beating

To get a rough estimate of the frequency of rat EHT effects in a non-selected group of drugs a chemical library was analyzed. NCEs were purchased from Maybridge and randomly chosen by respecting the rule of five [26]. 28 NCEs were tested at 0.1, 1, and 10 µM. Most NCEs had no effect on EHT contractility. Three NCEs prolonged T2 and one reduced force. In two additional cases EHTs stopped beating and didn’t react to electrical pacing (Supplemental Table 2). Overall, an effect on EHT contractility was observed in 21 % of the investigated NCEs.

## Discussion

Assessing the proarrhythmic risks of drugs remains a challenge in preclinical drug development. Current experimental models in preclinical toxicology determine the effect of NCEs on ion channels commonly involved in arrhythmogenic drug effects, particularly hERG channels [39], on electrophysiological surrogates of arrhythmias such as triangulation in Langendorff-perfused rabbit hearts [23, 24], on action potential duration in rabbit Purkinje fibers [15] or the QTc intervall in telemetrically surveyed dogs [13]. Newer models with higher throughput and/or a human cardiomyocyte context include measurements of heart rate in zebrafish [27], of Ca^2+^ transients in isolated guinea-pig [30] or human pluripotent stem cell (hPSC)-derived cardiomyocytes [5] or of electric field potentials in hPSC-cardiomyocytes [2]. The present study in rat EHTs is, to the best of our knowledge, the largest head-to-head comparison of proarrhythmic drugs performed so far. It showed that time of relaxation (T2) in rat EHTs is essentially insensitive to selective blockers of I_Kr_ or I_Ks_, but prolonged by inhibition of I_to_, combined full inhibition of I_Kr_ and I_Ks_ or combined inhibition of I_Ks_ and I_to_. This suggests that rat EHTs monitor mainly I_to_ and I_Ks_ effects of drugs. The I_to_-blocker 4AP also prolonged action potential duration and Ca^2+^ transients at T2-effective concentrations, indicating that, in this experimental model, T2 prolongation follow similar prolongations of repolarization and Ca^2+^ transients. The data suggest that, for drug screening purposes, T2 is a useful surrogate for time of repolarization in rat EHTs. The high percentage of known proarrhythmic drugs that induced concentration-dependent T2 prolongations, aftercontractions and/or irregular beating (group 1 and 2, 23/38 = 61 %) indicates that many clinically used drugs possess effects on I_to_ and/or I_Ks_ in addition to their well-characterized inhibitory action of I_Kr_ (hERG).

### Characterization of rat EHTs as a test system for testing proarrhythmic drugs

The underlying hypothesis of this study was that repolarization-inhibiting drugs prolong action potential duration and Ca^2+^ influx and thereby time of relaxation, a parameter easily assessable in EHT. EHTs are engineered three-dimensional cardiac tissue constructs in which cardiac cells are embedded in a fibrin matrix and, over 10–14 days, form a highly organized longitudinally oriented network [16]. The 24-well mini-EHTs system is designed for a robust, automated measurement of spontaneous or electrically stimulated contractile activity at a medium throughput scale. It is stable for weeks and has the advantage that measurements can be done at near-physiological conditions: 37 °C, steady state beating (not dying preparations such as isolated myocytes, papillary muscles, Purkinje fibers, and Langendorff hearts), auxotonically work-performing heart muscle constructs, and absence of invasive experimental interventions (e.g., patch clamp or microelectrodes). On the other hand, it is not well suited for measuring effects of drugs on action potentials, field potentials, or ion channels directly. We therefore performed several experiments to evaluate whether the relaxation time T2 is indeed a suitable surrogate of time of repolarization.

The following observations favor this assumption. (1) T2 is easily determined and highly reproducible (11 % SD, 0.77 % SEM, *n* = 221). (2) The duration of contraction and relaxation corresponds well with that of action potentials in rat EHTs and both were prolonged in the presence of 4AP as well as quinidine. Action potential duration (APD_90_) measured with sharp microelectrodes at 36 °C amounted to a mean of 113 ms, time to 90 % Ca^2+^ return to a mean of 124 ms at 37 °C, and T1 + T2 (at 80 % relaxation) amounted to a mean of 159 ms at 37 °C (this study and [16]). Thus, it is reasonable to assume that prolongations of repolarization affect T2. (3) Drugs with well-characterized actions on I_to_ [41], I_Kr_ [43], and I_Ks_ [36] had reproducible, concentration-dependent and reversible effects on T2. 4AP started to prolong T2 at 3 mM, which corresponds to 3-fold IC_50_ in adult rat cardiac myocytes [41] and confirms the prominent role of I_to_ for rodent heart repolarization [19]. The lack of effect of E-4031, dofetilide, ibutilide, and HMR-1556 even at 100-fold IC_50_ concentrations confirms the minor role of I_Kr_ and I_Ks_ for repolarization in rat cardiac myocytes [29]. Interestingly, however, the combined application of high concentrations of E-4031 and HMR-1556 markedly prolonged T2. Given the high selectivity of both drugs for I_Kr_ and I_Ks_ [36, 41], respectively, the data suggest that I_Kr_ and I_Ks_ can substitute for each other, but together play a role for repolarization in rat EHTs. The strong effect of HMR-1556 in the presence of 3 mM 4AP (and the lack of an E-4031 effect under this condition) suggests a greater role for I_Ks_ than I_Kr_. The necessary channel subunits are expressed in rat EHTs (KCNH2a, KCNQ1a, and KCNE1/E2 mRNAs are present in rat EHTs [16]).

Whereas all this argues for repolarization as an important parameter of relaxation time T2 in our system, it is obvious that other mechanisms have to be considered and that T2 prolongations alone do not prove effects on repolarization. Any effect on beating rate will affect T2 because of the well-known frequency-dependent acceleration of relaxation. Such effect could be excluded in case of quinidine and erythromycin (Supplemental Fig. 5), but may play a role in other cases. Drugs that directly affect intracellular Ca^2+^ handling could alter T2 independently of repolarization. Indeed, caffeine at a high concentration (5 mM) which enhances the open probability of RyR2 reduced beating rate and caused widening of contraction twitches (Supplemental Fig. 1). Thapsigargin, a selective inhibitor of SERCA, did not affect time of relaxation as such (3–30 nM), but aggravated the relaxation-slowing effect of 4AP (Supplemental Fig. 2). Another possibility to affect T2 independently of repolarization is an effect on myofilament Ca^2+^ sensitivity. Ca^2+^ sensitizers such as EMD 57033 [34] shift the force-pCa curve to the left and thereby prolong relaxation as recently shown in mouse EHTs [35]. The unexpected T2-prolongation under diltiazem may represent this mechanism. A study in isolated adult rat ventricular myocytes reported a mild myofilament Ca^2+^ sensitizing effect of diltiazem [9]. Such an effect may also explain the unexpected lack of significant negative inotropic effects of this compound at the highest tested concentration (10 µM) which was in contrast to verapamil. Conversely, epinephrine (Supplemental Fig. 3) and all other cAMP-dependent drugs increase the PKA-dependent phosphorylation of troponin I, myosin binding protein C and others and shift the curve to the right. This favors relaxation and abbreviates T2. Taken together, T2-prolongation of rat EHTs can be caused not only by blockers of repolarization (mainly I_to_, I_Ks_, very little contribution of I_Kr_), but also rate-slowing drugs, Ca^2+^ sensitizers or blockers of cAMP-dependent pathways such as carbachol (Supplemental Fig. 3), which needs to be considered in drug screening efforts.

The pronounced prolongation of relaxation at high concentrations of 4AP and aftercontractions were sensitive to both SEA0400 and JTV519 (Fig. 4), but relaxation remained significantly prolonged in their presence (~200 %). This finding is interesting as it suggests that the primary effect of 4AP, the inhibition of repolarization, has time-dependent (see time course in Fig. 4) secondary effects that affect time of relaxation. Very likely, this secondary effect corresponds to increased filling of the SR with Ca^2+^ with the final consequence of RyR2-mediated spontaneous Ca^2+^ release and NCX-mediated Ca^2+^ extrusion. The latter transport is electrogenic and contributes to prolonged depolarization and after depolarizations, visible in our system as marked prolongations of T2 and aftercontractions. An interesting speculation is that group 1 (Fig. 8) drugs directly interfere with RyR2 and/or NCX or SR function and therefore induce irregular beating, whereas group 2 drugs primarily affect repolarization and therefore prolong T2 before causing extra beats. On the other side, group 1 and 2 effects cannot be firmly separated because three group 1 drugs also caused T2 prolongations. Interestingly, tetracaine (also flecainide (0.5 µM); data not shown) did not mimic the effect of JTV519 (Supplemental Fig. 2), although both have inhibitory effects on RyR2-mediated Ca^2+^ release [18, 40]. Possibly, their main effect on I_Na_ which is not shared by JTV519 could explain the difference.

### Drug-induced relaxation slowing and beating irregularities in rat EHTs

We selected drugs according to a list published by Redfern and colleagues [31] that grouped drugs according to their proarrhythmic potential. 15/33 drugs with various degree of risk for TdP (Class I–IV) showed T2 prolongation or beating irregularities in our system (group 1 and 2). The percentage did not clearly differ between those that were withdrawn from the market for TdP (1/5) and those with only isolated TdP reports (6/13). In addition, T2-prolongation was observed in 6/14 drugs considered safe. This clearly indicates that the rat EHTs test does not sufficiently discriminate between high and low risk drugs, very likely due to the lack of I_Kr_ sensitivity.

Nevertheless, screening the large number of drugs revealed a number of interesting novel informations. (1) It is apparent that T2 prolongations and beating irregularities were seen mainly in those drugs which reach high FTPC in clinical use and were therefore tested at high absolute concentrations (compare concentration range of Group 1 and 2 drugs with group 3 and 4; Fig. 8). This confirms the general rule in pharmacology that drugs with low potency at their target have a higher chance of off-target toxicity than high affinity drugs. (2) Most effects were seen at 30–100-fold FTPC and higher. This is at the upper limit generally considered a critical safety margin [31] and point to a relatively low sensitivity of our assay. Rather than indicating simple non-specific effects (drugs such as sematilide and ciprofloxacin were tested at up to 300 µM) the data with E-4031, HMR-1556 and 4AP indicate that almost complete block of one or more currents is required to overcome safety mechanisms of repolarization in a relatively intact system such as the EHT. As such, the low sensitivity probably reflects the situation in vivo better than isolated cells [28]. Pharmacokinetic peculiarities and time-dependent effects likely add to high concentration requirements. For example, amiodarone is very lipophilic and accumulates in cells over time. With an apparent volume of distribution of 20–200 l/kg, the low FTPC of amiodarone (0.3 nM) is orders of magnitude lower than cellular concentrations in the steady state. (3) Concentrations in which group 1 and 2 drugs exerted their effects on EHTs were generally well above their IC_50_ for hERG (gray bars in Fig. 8). Notable exceptions are domperidone and phenytoin, where relaxation slowing occurred at concentrations below hERG IC_50_. Since E-4031 at high concentration markedly potentiated the effect of HMR-1556 without having an effect alone, the data suggest that inhibition of I_Kr_ may participate in the effect of group 1 and 2 drugs, but is not sufficient to fully explain them. Inhibition of other currents must come into play. We found reports of I_to_-inhibiting activity for 14 drugs (blue bars in Fig. 8). In four of these (imipramine, propafenone, quinidine, and clarithromycin) published IC_50_ values for hERG and I_to_ were at or below the threshold for T2 prolongation or irregularity, providing a likely mechanism of action. Moreover, we found reports of I_Ks_-inhibiting effects for 20 drugs (green bars in Fig. 8). In six of these cases (bepridil, thioridazine, propafenone, quinidine, cibenzoline, and sotalol), the IC_50_ was at or below the threshold for group 1 or 2 effects. It is interesting that quinidine at the T2-threshold concentration (100 µM) inhibits all three currents I_Kr_, I_to_, and I_Ks_. Other currents involved in rat cardiac repolarization such as I_K1_, I_ss_, and I_Kx_ [19] or I_KATP_ [4] and I_KNa_ are also potential targets, but not much is known about effects of the drugs investigated. Taken together, the data indicate that many clinically used drugs, some of them associated with TdP, others not, inhibit cardiac repolarizing currents in addition to I_Kr_. This supports recent data suggesting that combined channel block underlies clinical proarrhythmia [28].

### Miscellaneous effects on EHT function

Some drugs caused T1 prolongations (haloperidol, sertindole, diphenhydramine, and mefloquine), T2 acceleration (terfenadine) or negative inotropic effects (verapamil). Whereas the latter is the expected main effect of the drug, the mechanism of the other is unclear at present. T1 prolongations could indicate reduced conduction velocity in the EHT, a typical consequence of I_Na_ inhibition. However, pure I_Na_ blockers such as TTX or lidocaine did not prolong T1 (data not shown), arguing against this idea. An alternative may be an effect on gap junction conduction. At least mefloquine is known to inhibit numerous connexins (C×), including C × 43 [8]. T2 shortening could be due to accelerated repolarization by stimulation of K-currents or accelerated myofilament relaxation, but also due to an inhibition of I_Na_. In fact, terfenadine, which also shortened action potential duration in the SCREENIT test system (10 µM [23]), has recently been suggested to cause arrhythmias not by a TdP mechanism, but by its strong I_Na_-blocking activity [46].

Taken together, rat EHTs are not suitable as a general screening assay for proarrhythmic drug effects due to the small contribution of I_Kr_ for rat EHT repolarization. On the other hand, the assay is a simple and robust system for the analysis of non-hERG-related drug effects on cardiac function. Relaxation time was shown to be a particularly suited screening parameter, sensitive to drugs affecting cardiac repolarization, but also Ca^2+^ handling or myofilament function. The high fraction of drugs with known arrhythmogenic effects that prolonged relaxation or induced irregular beating in this hERG-insensitive system suggest that I_to_ and I_Ks_ effects add to the proarrhythmic risk of drugs and require further consideration.

### Electronic supplementary material

Below is the link to the electronic supplementary material.

Supplementary material 1 (DOCX 1814 kb)

## Appendix

EHTs were adopted from Redfern et al. 2003. Case reports (indicated by asterisk), estimated plasma concentration and IC50 values on Ito/IKs were obtained by a literature search.

^1^William S Redfern et al. 2003; *Cardiovasc Res*

^2^Daniel Scagliotti et al. 1982; *Am J Cardiol*

^3^Maasaki Yasuda et al. 2006; *Circ J*

^4^Martin Karch et al. 1997; *Herz*

^5^Toshihiro Ohata et al. 2010; *Thorac Surg*

^6^Joao V Vitola et al. 1998; *J Cardiovasc Electrophysiol*

^7^Hideyuki Kamochi et al. 1999; *Jpn Circ J*

^8^Franco Casazza et al. 1986; *G Ital Cardiol*

^9^Dagfinn AArskog and Asmund Reikvam 1992; *Tidsskr Nor Laegefore*

^10^Etienne Delacrétaz and Jürg Fuhrer 1999; *Clin Cardiol*

^11^Charlotte van Noord et al. 2010; *Drug Saf*

^12^Arthur J Moss and Joel Morganroth 1999; *Drug Saf*

^13^Michael W Brandriss et al. 1994; *Clin Infect Dis*

^14^Yigal Pinto et al. 1999; *Lancet*

^15^Juan M Nogales Asensio et al. 2007; *Int J Cardiol*

^16^KM Goel and RA Shanks; *Br Med J*

^17^Ngai-Shing Mok et al. 2005; *J Cardiovasc Electrophysiol*

^18^Winoc Fonteyne et al. 1996; *Clin Cardiol*

^19^John C DeToledo et al. 2001; *Epilepsia*

^20^Dan Tzivoni et al. 1981; *Arch Intern Med*

^21^John T Hii et al. 1991; *Pacing Clin Electrophysiol*

^22^Kwadwo Amankwa 2004; *Clin Pharmacol Ther*

^23^Wladyslaw Sinkiewicz 2006; *Pol Arch Med Wewn*

^24^Quian-Yong Liu et al. 1997; *Acta Pharmacol Sin*

^25^Ki-Wug Sung 2013; *Naunyn Schmiedebers Arch P*

^26^Dominique Abela 2010; *Birth Defects Res B Dev*

^27^Dong Zhang et al. 2011; *Acta Pharmacol Sin*

^28^Sean A Cahill et al. 2001; *J Cardiovasc Pharmacol*

^29^Pascale Gluais et al. 2003; *Fundam Clin Pharmacol*

^30^F Berger et al. 1989; *Naunyn Schmiedebers Arch P*

^31^L Xu et al. 2008; *Pharmazie*

^32^Pascale Gluais et al. 2004; *Eur J Pharmacol*

^33^Oscar Casis et al. 1998; *J Cardiovasc Pharmacol*

^34^Imju Jeong et al. 2013; *Brain Res*

^35^Aaref Badshah et al. 2009; *Am J Med Sci*

^36^Yoshihiro Yumoto et al. 2004; *J Cardiovasc Pharmacol*

^37^Benoit Drolet et al. 1999; *J Pharmacol Exp Ther*

^38^Dao Wu Wang et al. 1996; *J Moll Cell Cardiol*

^39^Ling-Ping Lai et al. 1999; *J Biomed Sci*

^40^Joseph J Salata et al. 1995; *Circ Res*

^41^Jisheng Kang et al. 2001; *J Pharmacol Exp Ther*

^42^Jisheng Kang et al. 2000; *Eur J Pharmacol*

^43^Stephen E Jones et al. 1998; *Br J Pharmacol*

^44^Christiaan C Veerman et al. 2013; *Circ Arrhythm Electrophysio*l

^45^Edward Carmeliet 1998; *Br J Pharmacol*

## References

[1] Ackerman MJ, Mohler PJ (2010). Defining a new paradigm for human arrhythmia syndromes: phenotypic manifestations of gene mutations in ion channel- and transporter-associated proteins. Circ Res.

[2] Braam SR, Tertoolen L, van de Stolpe A, Meyer T, Passier R, Mummery CL (2010). Prediction of drug-induced cardiotoxicity using human embryonic stem cell-derived cardiomyocytes. Stem Cell Res.

[3] Brown AM (2009). High throughput functional screening of an ion channel library for drug safety and efficacy. Eur Biophys J.

[4] Burley DS, Cox CD, Zhang J, Wann KT, Baxter GF (2014). Natriuretic peptides modulate ATP-sensitive K(+) channels in rat ventricular cardiomyocytes. Basic Res Cardiol.

[5] Cerignoli F, Charlot D, Whittaker R, Ingermanson R, Gehalot P, Savchenko A, Gallacher DJ, Towart R, Price JH, McDonough PM, Mercola M (2012). High throughput measurement of Ca^2+^ dynamics for drug risk assessment in human stem cell-derived cardiomyocytes by kinetic image cytometry. J Pharmacol Toxicol Methods.

[6] Christ T, Galindo-Tovar A, Thoms M, Ravens U, Kaumann AJ (2009). Inotropy and L-type Ca2^+^ current, activated by beta1- and beta2-adrenoceptors, are differently controlled by phosphodiesterases 3 and 4 in rat heart. Br J Pharmacol.

[7] Crocini C, Arimura T, Reischmann S, Eder A, Braren I, Hansen A, Eschenhagen T, Kimura A, Carrier L (2013). Impact of ANKRD1 mutations associated with hypertrophic cardiomyopathy on contraction parameters of engineered heart tissue. Basic Res Cardiol.

[8] Cruikshank SJ, Hopperstad M, Younger M, Connors BW, Spray DC, Srinivas M (2004). Potent block of C × 36 and C × 50 gap junction channels by mefloquine. Proc Natl Acad Sci USA.

[9] Davis J, Wen H, Edwards T, Metzger JM (2008). Allele and species dependent contractile defects by restrictive and hypertrophic cardiomyopathy-linked troponin I mutants. J Mol Cell Cardiol.

[10] Echt DS, Liebson PR, Mitchell LB, Peters RW, Obias-Manno D, Barker AH, Arensberg D, Baker A, Friedman L, Greene HL (1991). Mortality and morbidity in patients receiving encainide, flecainide, or placebo. The cardiac arrhythmia suppression trial. N Engl J Med.

[11] EMA (2005) The nonclinical evaluation of the potential for delayed ventricular repolarization (Qt interval prolongation) by human pharmaceuticals S7B 16237859

[12] FDA (2005) Guidance for industry S7B nonclinical evaluation of the potential for delayed ventricular repolarization (QT interval prolongation) by human pharmaceuticals 16237859

[13] Fossa AA, Depasquale MJ, Raunig DL, Avery MJ, Leishman DJ (2002). The relationship of clinical QT prolongation to outcome in the conscious dog using a beat-to-beat QT-RR interval assessment. Pharmacology.

[14] Gintant GA (2008). Preclinical Torsades-de-Pointes screens: advantages and limitations of surrogate and direct approaches in evaluating proarrhythmic risk. Pharmacol Ther.

[15] Gintant GA, Limberis JT, McDermott JS, Wegner CD, Cox BF (2001). The canine Purkinje fiber: an in vitro model system for acquired long QT syndrome and drug-induced arrhythmogenesis. J Cardiovasc Pharmacol.

[16] Hansen A, Eder A, Bönstrup M, Flato M, Mewe M, Schaaf S, Aksehirlioglu B, Schwoerer AP, Schwörer A, Uebeler J, Eschenhagen T (2010). Development of a drug screening platform based on engineered heart tissue. Circ Res.

[17] Haverkamp W, Breithardt G, Camm aJ, Janse MJ, Rosen MR, Antzelevitch C, Escande D, Franz M, Malik M, Moss a, Shah R (2000). The potential for QT prolongation and pro-arrhythmia by non-anti-arrhythmic drugs: clinical and regulatory implications. Report on a Policy Conference of the European Society of Cardiology. Cardiovasc Res.

[18] Hilliard FA, Steele DS, Laver D, Yang Z, Le SJ, Chopra N, Piston DW, Huke S, Knollmann BC (2010). Flecainide inhibits arrhythmogenic Ca^2+^ waves by open state block of ryanodine receptor Ca^2+^ release channels and reduction of Ca^2+^ spark mass. J Mol Cell Cardiol.

[19] Himmel HM, Wettwer E, Li Q, Ravens U (1999). Four different components contribute to outward current in rat ventricular myocytes Four different components contribute to outward current in rat ventricular myocytes. Am J Physiol.

[20] Hirt MN, Sörensen NA, Bartholdt LM, Boeddinghaus J, Schaaf S, Eder A, Vollert I, Stöhr A, Schulze T, Witten A, Stoll M, Hansen A, Eschenhagen T (2012). Increased afterload induces pathological cardiac hypertrophy: a new in vitro model. Basic Res Cardiol.

[21] Hoffmann P, Warner B (2006). Are hERG channel inhibition and QT interval prolongation all there is in drug-induced torsadogenesis? A review of emerging trends. J Pharmacol Toxicol Methods.

[22] Hondeghem LM, Carlsson L, Duker G (2001). Instability and triangulation of the action potential predict serious proarrhythmia, but action potential duration prolongation is antiarrhythmic. Circulation.

[23] Hondeghem LM, Dujardin K, Hoffmann P, Case IA, Of S (2011). Drug-induced QT C prolongation dangerously underestimates proarrhythmic potential : lessons from terfenadine. Baseline.

[24] Hondeghem LM, Hoffmann P (2003). Blinded test in isolated female rabbit heart reliably identifies action potential duration prolongation and proarrhythmic drugs: importance of triangulation, reverse use dependence, and instability. J Cardiovasc Pharmacol.

[25] Laverty H, Benson C, Cartwright E, Cross M, Garland C, Hammond T, Holloway C, McMahon N, Milligan J, Park B, Pirmohamed M, Pollard C, Radford J, Roome N, Sager P, Singh S, Suter T, Suter W, Trafford a, Volders P, Wallis R, Weaver R, York M, Valentin J (2011). How can we improve our understanding of cardiovascular safety liabilities to develop safer medicines?. Br J Pharmacol.

[26] Lipinski CA, Lombardo F, Dominy BW, Feeney PJ (2001). Experimental and computational approaches to estimate solubility and permeability in drug discovery and development settings. Adv Drug Deliv Rev.

[27] Milan DJ, Peterson TA, Ruskin JN, Peterson RT, Calum A (2003). Drugs that induce repolarization abnormalities cause bradycardia in zebrafish. Circulation.

[28] Nalos L, Varkevisser R, Jonsson MKB, Houtman MJC, Beekman JD, van der Nagel R, Thomsen MB, Duker G, Sartipy P, de Boer TP, Peschar M, Rook MB, van Veen TAB, van der Heyden MAG, Vos MA (2012). Comparison of the IKr blockers moxifloxacin, dofetilide and E-4031 in five screening models of pro-arrhythmia reveals lack of specificity of isolated cardiomyocytes. Br J Pharmacol.

[29] Nerbonne JM, Kass RS (2005). Molecular physiology of cardiac repolarization. Physiol Rev.

[30] Qian J-Y, Guo L (2010). Altered cytosolic Ca^2+^ dynamics in cultured Guinea pig cardiomyocytes as an in vitro model to identify potential cardiotoxicants. Toxicol In Vitro.

[31] Redfern WS, Carlsson L, Davis aS, Lynch WG, MacKenzie I, Palethorpe S, Siegl PKS, Strang I, Sullivan aT, Wallis R, Camm aJ, Hammond TG (2003). Relationships between preclinical cardiac electrophysiology, clinical QT interval prolongation and torsade de pointes for a broad range of drugs: evidence for a provisional safety margin in drug development. Cardiovasc Res.

[32] Regan CP, Cresswell HK, Zhang R, Lynch JJ (2005) Novel method to assess cardiac electrophysiology in the rat: characterization of standard ion channel blockers. J Cardiovasc Pharmacol 46:68–75. doi:10.1097/01.fjc.0000162774.86780.9d 10.1097/01.fjc.0000162774.86780.9d 15965357

[33] Schaaf S, Shibamiya A, Mewe M, Eder A, Stöhr A, Hirt MN, Rau T, Zimmermann W-H, Conradi L, Eschenhagen T, Hansen A (2011). Human engineered heart tissue as a versatile tool in basic research and preclinical toxicology. PLoS One.

[34] Solaro RJ, Gambassi G, Warshaw DM, Keller MR, Spurgeon Ha, Spurgeon Ha, Beier N, Lakatta EG (1993). Stereoselective actions of thiadiazinones on canine cardiac myocytes and myofilaments. Circ Res.

[35] Braam SR, Tertoolen L, van de Stolpe A, Meyer T, Passier R, Mummery CL (2010). Prediction of drug-induced cardiotoxicity using human embryonic stem cell-derived cardiomyocytes. Stem Cell Res.

[36] Stöhr A, Friedrich FW, Flenner F, Geertz B, Eder A, Schaaf S, Hirt MN, Uebeler J, Schlossarek S, Carrier L, Hansen A, Eschenhagen T (2013). Contractile abnormalities and altered drug response in engineered heart tissue from Mybpc3-targeted knock-in mice. J Mol Cell Cardiol.

[37] Thomas GP, Gerlach U, Antzelevitch C (2003). HMR 1556, a potent and selective blocker of slowly activating delayed rectifier potassium current. J Cardiovasc Pharmacol.

[38] Thomsen MB, Matz J, Volders PGa, Vos MA (2006). Assessing the proarrhythmic potential of drugs: current status of models and surrogate parameters of torsades de pointes arrhythmias. Pharmacol Ther.

[39] Usdin S, Haan K (2003) Chapter 11. In: The QT mandate, BioCentury, Washington, pp A1–A5

[40] Vandenberg JI, Walker BD, Campbell TJ (2001). HERG K+ channels: friend and foe. Trends Pharmacol Sci.

[41] Venetucci LA, Trafford AW, O’Neill SC, Eisner DA (2007). Na/Ca exchange: regulator of intracellular calcium and source of arrhythmias in the heart. Ann N Y Acad Sci.

[42] Volk T, Nguyen TH, Schultz JH, Ehmke H (1999). Relationship between transient outward K+ current and Ca^2+^ influx in rat cardiac myocytes of endo- and epicardial origin. J Physiol.

[43] Wible BA, Kuryshev YA, Smith SS, Liu Z, Brown AM (2008). An ion channel library for drug discovery and safety screening on automated platforms. Assay Drug Dev Technol.

[44] Zhou Z, Gong Q, Ye B, Fan Z, Makielski JC, Robertson GA, January CT (1998). Properties of HERG channels stably expressed in HEK 293 cells studied at physiological temperature. Biophys J.

[45] Zimmermann WH, Fink C, Kralisch D, Remmers U, Weil J, Eschenhagen T (2000). Three-dimensional engineered heart tissue from neonatal rat cardiac myocytes. Biotechnol Bioeng.

[46] Zimmermann W-H, Schneiderbanger K, Schubert P, Didié M, Münzel F, Heubach JF, Kostin S, Neuhuber WL, Eschenhagen T (2002). Tissue engineering of a differentiated cardiac muscle construct. Circ Res.

[47] Lu HR, Hermans AN, Gallacher DJ (2012). Does terfenadine-induced ventricular tachycardia/fibrillation directly relate to its QT prolongation and Torsades de Pointes?. Br J Pharmacol.

