# Supplementary material for: Effects of proarrhythmic drugs on relaxation time and beating pattern in rat engineered heart tissue
Source: Basic Res Cardiol. 2014 Sep 11;109(6):436. doi: 10.1007/s00395-014-0436-7 (PMC4160570; doi:10.1007/s00395-014-0436-7)
Supplement: Supplementary file 1 — Supplementary material 1 (DOCX 1814 kb) [file 395_2014_436_MOESM1_ESM.docx]

**SUPPLEMENTAL MATERIAL**

**Supplemental Methods**

*Cell Isolation and EHT generation*

Unpurified heart cells were isolated from neonatal Wistar rats (postnatal day 0 to 3) by a fractionated DNase/Trypsin digestion protocol as previously described [6]. The resulting cell population was immediately subjected to EHT generation. Experimental procedures were reviewed and approved by Ethics Committee, University Hamburg.

To generate EHTs a reconstitution mix was prepared on ice as follows (final concentration): 4.1x10^6^cells/ml and 5 mg/ml bovine fibrinogen (stock solution: 200 mg/ml plus aprotinin 0.5 µg/mg fibrinogen in NaCl 0.9%, Sigma F4753). 2xDMEM (20% horse serum, 4% chick embryo extract, 2% Penicillin/Streptomycin) was added to match the volumes of fibrinogen and thrombin stock to ensure isotonic conditions. Casting molds were prepared by placing the teflon spacer in 24-well culture dishes and adding 1.6 ml 2% agarose (Invitrogen 15510-027) in PBS per well. After agarose solidification the spacer were removed and silicon post racks (Silitec GmbH & Co. KG, Weiler-Simmerberg, Germany) were placed onto the dishes with pairs of posts reaching into each casting mold. For each EHT 97 µl reconstitution mix was mixed briefly with 3.0 µl thrombin (stock solution: 100 U/ml, final concentration: 3 U/ml, Biopur BP 11-10-1104) and pipetted into the agarose slot (LxWxD 12x3x4 mm). After 2 h incubation (37 °C, 7% CO_2_, 40% O_2_), fibrin blocks were polymerized and could be transferred into a new 24-well cell culture plate filled with medium (DMEM (Biochrom F0415) with 10% horse serum (Gibco 26050), 2% chick embryo extract, 1% Penicillin/Streptomycin (Gibco 15140), insulin (10 µg/ml, Sigma-Aldrich I9278), aprotinin (33 µg/ml, Sigma-Aldrich A1153)) and kept for up to three weeks. Medium was changed every other day.

*Measurement of contractile parameter*

Contractile parameters were evaluated by video-optical recordings as previously described [1]. The setup for video-optical analyses consisted of a cell incubator-like unit, in which gas conditions, humidity and temperature could be controlled. This device was equipped with a glass roof for monitoring purposes. A Basler CCD-camera (Type A 602f-2) was attached to a XYZ-device (IAI Corporation) and positioned above the glass roof in a PC-controlled manner. Light-emitting diodes (LEDs) were placed underneath the cell culture dish. Illumination of a single LED was synchronized with the video-optical recording procedure in order to minimize heating of the cell culture medium by LED waste heat. For the calculation of contractile parameters a customized software package developed by Consulting Team Machine Vision (ctmv.de; Pforzheim, Germany) was used. This software is based on figure recognition and is able to identify the EHT’s shape in a fully automated manner. Measuring points were then placed at the top and bottom end of the contracting muscle strip. Due to the contraction, the silicone posts are deflected and this deflection (delta value of post distance) is determined and recorded by the software over time. Based on post geometry, elastic modulus of the silicone (1.7 MPa) and post deflection, the developed force was calculated based on a published equation [5]. The recorded contractions are filtered and identified as such by predefined peak criteria (e.g. threshold value, minimal force and minimum relaxation). Besides average force, the software calculates values for frequency, fractional shortening, contraction- and relaxation time (bpm, T1 and T2, respectively). T1 and T2 were determined at 20% of peak maximum. Reports with an overview of the environmental condition (temperature, gas, humidity) in the incubator-like unit plus all calculated parameters are automatically generated after each run. The effort to analyse a 24-well plate with contracting muscle strips is limited to defining the XYZ-coordinates for each well before starting a series of measurements.

*Drug screening – video optical analysis*

Drugs were chosen according to the Redfern-list [3]. In this publication, 52 drugs were listed and grouped into five categories ranked by their proarrhythmic potential. The first group encompasses drugs with the highest potential for proarrhythmic effects, the fifth those ones assumed to be save in humans. 46 of the commercially available drugs were incubated with EHTs. Previous experiments indicated that EHTs reached their force-plateau at day 14 and forces remained stable at least until day 21. Accordingly, all measurements were performed in between this period of time. Moreover, initial experiments indicated that the cell culture medium can have marked influence on the measurement. For example, horse serum (10%) and chick embryo extract (2%) in the standard EHT medium exert plasma protein binding of the drugs. Furthermore, we observed that fresh medium protected EHTs from proarrhythmic or relaxation-prolonging effects of drugs. We therefore developed a standard operation procedure with fresh serum-free DMEM (Biochrom F04115), supplemented with 10 mM HEPES for pH-steadiness and pre-incubated at 37 °C, 40% O_2_, 7% CO_2_, 90% humidity for 2 h. Measurements were done routinely one day after feeding with standard EHT medium. The drugs were analyzed in 3 different concentrations (45 min each, cumulative, 1-100 x free therapeutic plasma concentration [FTPC]). For a detailed list of all tested drugs see supplement Table 1. Prior to each measurement, 50 nM epinephrine (Sigma E4643) was added to each well to simulate physiological conditions and enhance the likelihood of contractile activity within the 60 s recording time. Drugs were added under sterile conditions. After 45 min incubation, EHTs were transferred to the video optical system. Due to a sequential mode of measurement, incubation time varied from the first to the last EHT. Nevertheless, incubation was performed for at least 45 min, which showed in initial experiments enough time to exhibit drug actions. Possible effects of the solvents DMSO, acidic water and ethanol were tested on EHTs at the highest concentrations employed. None of them exerted significant effects on EHT contractility parameters over a period of up to 75 min.

*Measurements under perfusion and electrical stimulation, Ca^2+^-transients*

To differentiate between effects on beating rate and relaxation and to analyse intracellular Ca^2+^-transients in addition to force, two drugs were additionally analysed under pacing at defined beating rate using a novel modified IonOptix (IonOptix Corporation, Milton, MA, USA) setup. This custom-made setup allowed continuous electrical stimulation and perfusion of EHTs during the measurement. The setup consisted of an inverted microscope, a temperature- and O_2_/CO_2_-controlled chamber for the 24-well EHT plate mounted on the microscope stage, a flow rate-controlled perfusion system, platinum wire electrodes for field stimulation and a video camera and software (both IonOptix) for the evaluation of contractile activity (edge detection mode). Experiments were done under a flow rate of approximately 4 ml/min (per well) and stimulation at 2-4 Hz and 7 V. Measurements were done in a modified Tyrode’s solution (mM: NaCl 120, KCl 5.4, MgCl_2_ 1.0, CaCl_2_ 1.8, NaH_2_PO_4_ 0.4, NaHCO_3_ 22.6, glucose 5.0, Na_2_EDTA 0.05, ascorbic acid 0.3). EHTs were equilibrated for 20 min in Tyrode’s solution (baseline) and then exposed to the respective drug for up to 20 min. Reversibility of drug effects was determined after 20 min perfusion in drug-free Tyrode’s solution.

For measurement of intracellular Ca^2+^-transients, EHTs were incubated in Tyrode’s solution containing 10 µM Fura2-AM (Invitrogen F1221) and the non-ionic detergent Cremophor EL (0.0075%; Sigma C5135) for 2 h at 37 °C. After washing the EHTs for 10 min in Tyrode’s solution, measurements under perfusion and stimulation of Ca^2+^-transients were performed in the same way as for force of contraction, but signals were evaluated by a 10x objective (instead of 1.25x for force) and a photomultiplier.The ratio of the emission intensity of light (510 nm) from EHTs alternatively excited at 340 and 380 nm (F340/380 ratio) was used as an index of cytosolic Ca^2+^-concentration. Data were evaluated either with the IonOptix software or a customized software [4] or manually when peaks were severely deformed.

**Supplemental Tables**

**Supplemental Table 1:** All drugs, except of quinidine (Carl-Roth) and SEA0400 (Endotherm), were purchased from Sigma-Aldrich and solved in DMSO (apart from 4-aminopyridine (medium), ciprofloxacin (acidic water) and tamoxifen (ethanol)).

| Drug | Cat.-No. | Class | LD_50_ |
| --- | --- | --- | --- |
| 4-aminopyridine (4AP) | A78403 | I_t0_-inhibitor |  |
| Amiodarone | A8423 | Antiarrhythmic class III | >3.000 mg∙kg^-1^ (rat, p. o.) |
| Amitryptiline | A8404 | Tricyclic antidepressant |  |
| Aprindine | A7606 | Antiarrhythmic class I_C_ |  |
| Astemizole | A2861 | Antihistamine | 35 mg∙kg^-1^ (mouse, i. v.) |
| Bepridil | B5016 | Antiarrhythmic class IV |  |
| Cetirizine | 89126 | Antihistamine | 365 mg∙kg^-1^ (rat, p. o.) |
| Chlorpheniramine | C3025 | Antihistamine | 118 mg∙kg^-1^ (rat, p. o.) |
| Cibenzoline | C1618 | Antiarrhythmic class I_A_ |  |
| Ciprofloxacin | 17850 | Antibiotic | 122 mg∙kg^-1^ (mouse, i. v.) |
| Cisapride | C4740 | Gastroprokinetic agent | 4.166 mg∙kg^-1^ (rat, p. o.) |
| Clarithromycin | C9742 | Antibiotic | 1.270 mg∙kg^-1^ (rat, p. o.) 850 mg∙kg^-1^ (mouse, i. p.) |
| Desipramine | D3900 | Tricyclic antidepressant | 740 mg∙kg^-1^ (mouse, p. o.) |
| Diltiazem | D2521 | Calcium channel blocker |  |
| Diphenhydramine | D3630 | Antihistamine | 390 mg∙kg^-1^ (rat, p. o.) |
| Disopyramide | D6035 | Antiarrhythmic class I_A_ | 333 mg∙kg^-1^ (rat, p. o.) |
| D,L-sotalol | S0278 | Antiarrhythmic class II | 790 mg∙kg^-1^ (mouse, i. p.) |
| Dofetilide | PZ0016 | Antiarrhythmic class III |  |
| Domperidone | D122 | Antiemetic | 5.243 mg∙kg^-1^ (rat, p. o.) 46,5 mg∙kg^-1^ (mouse, i. v.) |
| E4031 | M5060 | I_Kr_-inhibitor |  |
| Ebastine | E9531 | Antihistamine |  |
| Erythromycin | 856193 | Antibiotic | 4.600 mg∙kg^-1^ (rat, p. o.) 2.580 mg∙kg^-1^ (mouse, p. o.) |
| Fexofenadine | F9427 | Antihistamine |  |
| Flecainide | F6777 | Antiarrhythmic class I_C_ | 1.346 mg∙kg^-1^ (rat, p. o.) |
| Fluoxetine | F132 | Antidepressant | 825 mg∙kg^-1^ (rat, p. o.)  87,5 mg∙kg^-1^ (mouse, i. p.) |
| Haloperidol | H1512 | Antipsychotic | 128 mg∙kg^-1^ (rat, p. o.)  30 mg∙kg^-1^ (mouse, i. p.) |
| HMR-1556 |  | I_Ks_-inhibitor |  |
| Ibutilide | I9910 | Antiarrhythmic class III |  |
| Imipramine | I7379 | Tricyclic antidepressant | 250 mg∙kg^-1^ (rat, p. o.)  21 mg∙kg^-1^ (mouse, i. v.) |
| JTV519 | - | RyR2-stabilizer |  |
| Ketoconazole | K1003 | Fungicide | 166 mg∙kg^-1^ (rat, p. o.) |
| Loratadine | L9664 | Antihistamine | >5 g∙kg^-1^ (rat, p. o.) |
| Mefloquine | M2319 | Antiprotozoal agent | 880 mg∙kg^-1^ (rat, p. o.) |
| Mibefradil | M5441 | Antiarrhythmic class IV |  |
| Moxifloxacin | 32477 | Antibiotic | 1320 mg∙kg^-1^ (rat, p.o.) |
| Nifedipine | N7634 | Antihypertensive drug | 1.022 mg∙kg^-1^ (rat, p. o.) 185 mg∙kg^-1^ (mouse, i. p.) |
| Nitrendipine | N0516 | Antihypertensive drug | 2.540 mg∙kg^-1^ (mouse, p. o.) |
| Phenytoin | P4007 | Anticonvulsive drug/ Antiarrhythmic | 1.635 mg∙kg^-1^ (rat, p. o.) 100 mg∙kg^-1^ (mouse, i. p.) |
| Pimozide | P1793 | Antipsychotic drug | 288 mg∙kg^-1^ (mouse, p. o.) |
| Procainamide | P9391 | Antiarrhythmic class I_A_ | 1.950 mg∙kg^-1^ (rat, p. o.) |
| Propafenone | P4670 | Antiarrhythmic class I_C_ |  |
| Quinidine | 3348.1 | Antiarrhythmic class I_A_ |  |
| Risperidone | R3030 | Atypicalantipsychotic | 56,6 mg∙kg^-1^ (rat, p. o.) |
| SEA0400 | - | NCX-inhibitor |  |
| Sematilide | S0323 | Antiarrhythmic |  |
| Sertindole | S8072 | Antipsychotic | 420 mg∙kg^-1^ (mouse, i. p.) |
| Sparfloxacin | 56968 | Antibiotic |  |
| Tamoxifen | T5648 | Estrogenreceptorantagonist | 4.100 mg∙kg^-1^ (rat, p. o.) |
| Terfenadine | T9652 | Antihistamine | 5.000 mg∙kg^-1^ (mouse, p. o.) |
| Terodiline | T4577 | Antispasmotic |  |
| Tetracaine | T7383 | Antiarrhythmic class I_B_, RyR2-inhibitor |  |
| Tetrodotoxin | T8024 | I_Na_-inhibitor |  |
| Thapsigargin | T9033 | SERCA-inhibitor |  |
| Thioridazine | T9025 | Neuroleptic drug |  |
| Verapamil | 381195 | Calcium channel blocker | 163 mg∙kg^-1^ (rat, p. o.) |

| **NCE** | **Effect** |
| --- | --- |
| AW 00174 | - |
| AW 00180 | - |
| BTB 06225 | - |
| BTB 06226 | - |
| BTB 12144 | - |
| CD 09821 | * |
| CD 09829 | * |
| CD 10434 | - |
| GP 0210 | - |
| HTS 01892 | - |
| HTS 02410 | T2↑ |
| HTS 03564 | - |
| HTS 04977 | T2↑ |
| HTS 05264 | T2↑ |
| HTS 05819 | - |
| HTS 08362 | - |
| HTS 06400 | - |
| HTS 12320 | - |
| HTS 13511 | Force↓ |
| HTS 13515 | - |
| HTS 13518 | - |
| JFD 03976 | - |
| KM 02276 | - |
| KM 02284 | - |
| RH 0088 | - |
| RJF 02181 | - |
| S 11485 | - |
| SEW 03313SC | - |

**Supplemental Figures**

**Table 2: Analysis of new chemical entities (NCEs) on EHT contractility.**NCEs were purchased from Maybridge Inc. and randomly chosen by respecting the rule of five [2]. All NCEs were tested in three different concentrations (0.1, 1, 10 µM). Most of them had no effect on EHT contractility. Two NCEs caused a total arrest of beating even under electrical stimulation (*).


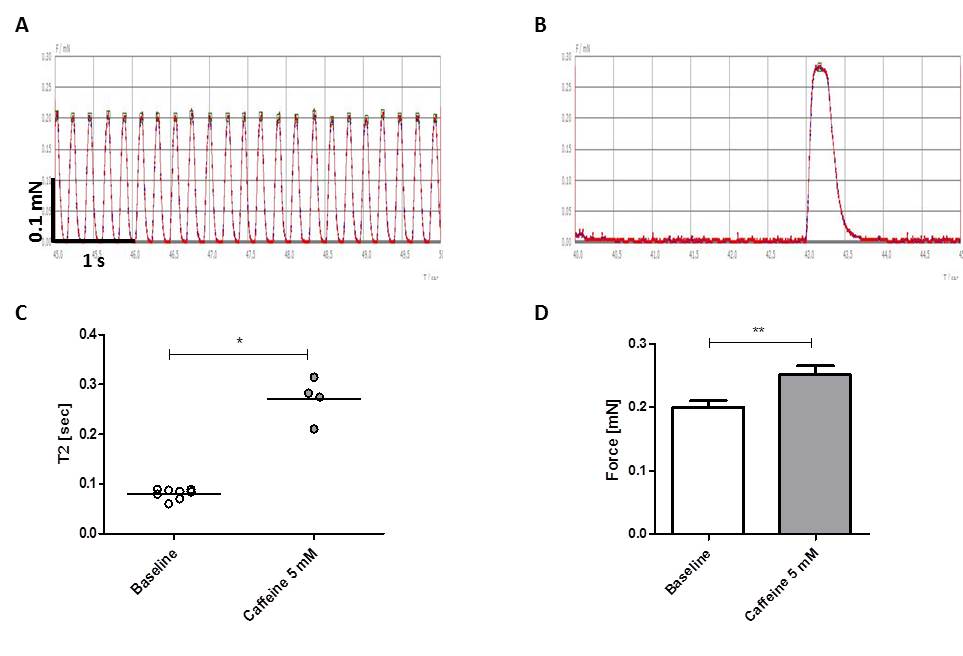


**Supplemental Figure 1: Effect of caffeine on EHT contraction. A** Original contraction recording under baseline condition and **B** after caffeine administration. **C** Statistical evaluation of relaxation time (T2), **D** of force. Each point represents one EHT. X-axes in A, B: time in seconds; y-axes: force in mN; y-axis in C: force in mN; y-axis in D: T2 in seconds; *p<0.05, **p<0.003 vs. baseline, Student’s t-test.

**Supplemental Figure 2: Effect of blockers of Ca^2+^-currents, fast Na^+^ currents or sarcoplasmic Ca^2+^ uptake on 4AP-induced prolongations of relaxation (T2) in spontaneously beating EHTs**. Baseline indicates pre-drug values. EHTs were either incubated for 45 min without drug (time control, TC) or with 4-aminopyridine (4AP; 10 mM) in the absence or presence of different concentrations of verapamil (**A**), tetracaine (**B**), tetrodotoxin (**C**) or thapsigargin (**D**). Note different scales and interruption of the y-axis. The compounds either did not affect T2-prolongation (verapamil) or augmented the 4AP-effect (thapsigargin, tetrodotoxin, tetracaine); *p<0.05, **p<0.003, ***p<0.0001 vs. baseline, one-way ANOVA + Dunnett’spost test).

**A**

**D**

**C**

**B**


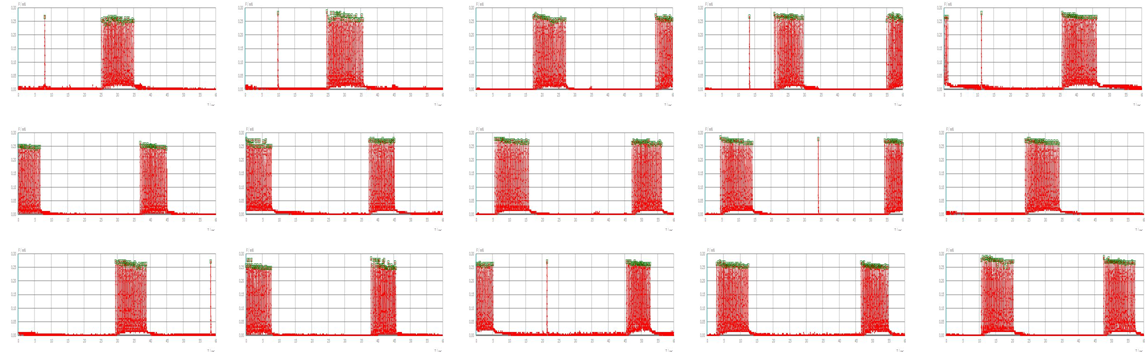


60 s

20

21

22

23

24

26

27

28

29

25

19

18

17

16

15

14

13

12

11

10

5

9

8

7

6

4

3

2

1

0

**A**

**C** Epinephrine 5 Hz

**B** Baseline 4 Hz


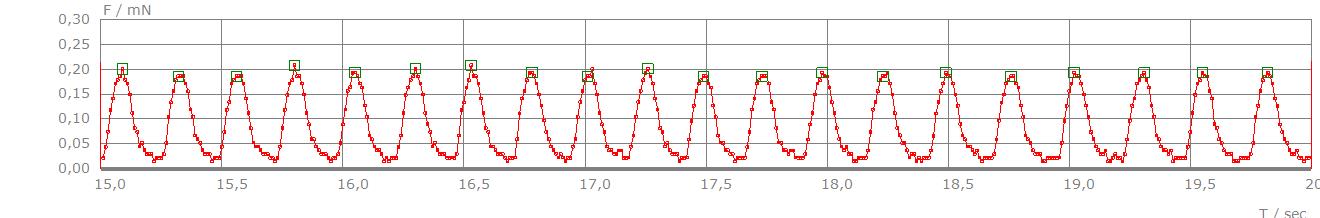

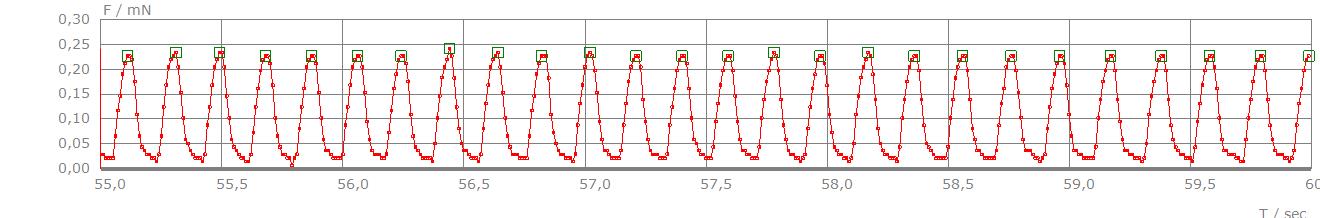


1 s

1 s

**D** Carbachol 4.4 Hz

n=7

n=7


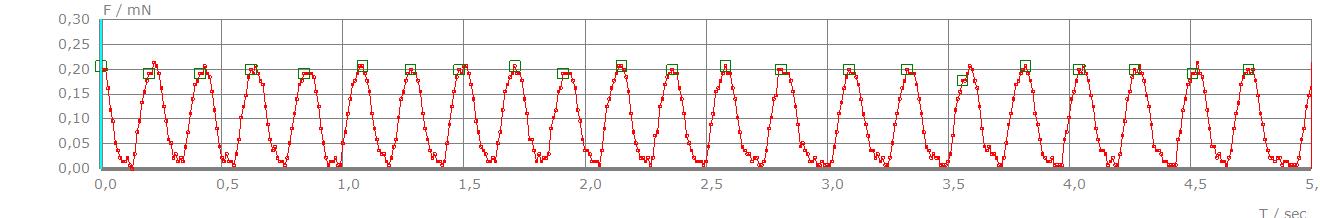


1 s

n=3

**Supplemental Figure 3:** Characteristic burst-like contraction pattern of rat EHTs. A Original recordings of repeated 1 min measurements of a single EHT over a period of 29 min. B-D Representative contractions at higher temporal resolution (5 s each) in the absence of drugs (B) and the presence of 50 nM epinephrine (C) or epinephrine plus 1 µM carbachol (D). Note that the contraction pattern was quite constant within the recording period with ~2 bursts/min, stable twitch force in the burst and a beating frequency in the bursts of 4.4-4.6 Hz. Epinephrine caused an increase in beating frequency to 5-5.6 Hz, associated with small increases in twitch force and decreases in contraction (T1) and relaxation time (T2). All effects were almost completely reversed by the addition of carbachol (1 µM). Under carbachol some of the EHTs (n=3/7) stopped spontaneous beating.

**Supplemental Figure 4: Original contraction recordings in the absence (baseline) and presence of proarrhythmic drugs representative for group 1 (A) and 2 (B).** **A** shows an irregular beating pattern induced by imipramine, **B** a sparfloxacin-induced prolongation of relaxation time (T2) associated with a marked reduction of beating rate and an increase in twitch force (x-axes: force in millinewton; y-axes: time in seconds).

Wash2

Wash 1

Q20 min

Q15 min

Q10 min

Control

A

Quinidine (Q) 100 µM

B

Erythromycin (E) 1 mM

E15 min

Wash 1

E20 min

E10 min

Wash 2

Control


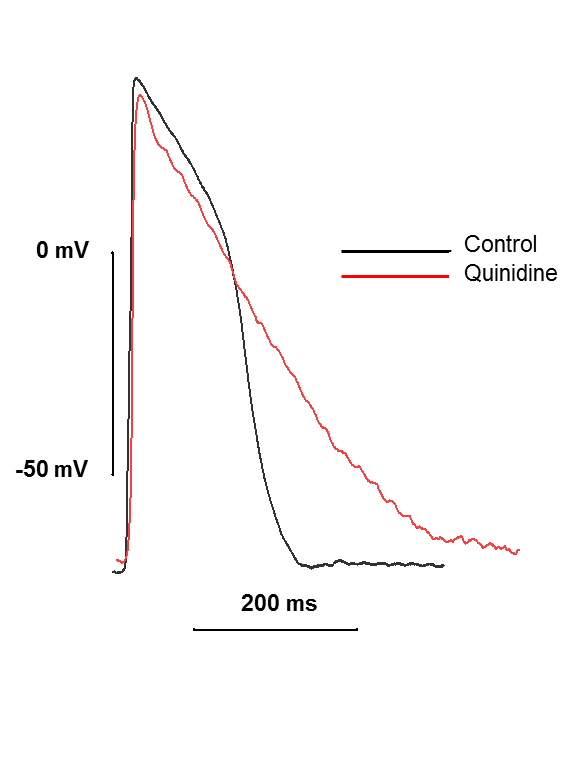


D

C

**Supplemental Figure 5:** Time course of the effect of high concentrations of quinidine (100 µM, A) on force and action potential duration (APD_90_) or erythromycin (1 mM, B) on force measured in electrically stimulated and continuously perfused EHTs (4 Hz for force, 5 Hz for APD_90_). Note widening of contraction twitch and reduction in twitch amplitude (Q 10 min, E15 min). Under quinidine, the beating pattern became regular again, but with enlarged peaks. C Average peaks at different incubation time point of quinidine, shortly before the EHT failed to capture and started to beat with an autonomous rhythm. In both cases the effects were fully reversible. D Average action potentials (APD_90_) in the presence (red line) and absence (black line) of quinidine (100 µM), showing a quinidine-induced increase in APD_90_.

**Supplemental References**

1. Hansen A, Eder A, Bönstrup M, Flato M, Mewe M, Schaaf S, Aksehirlioglu B, Schwoerer AP, Schwörer A, Uebeler J, Eschenhagen T (2010) Development of a drug screening platform based on engineered heart tissue. *Circ Res* 107:35–44 doi:10.1161/CIRCRESAHA.109.211458

2. Lipinski CA, Lombardo F, Dominy BW, Feeney PJ (2001) Experimental and computational approaches to estimate solubility and permeability in drug discovery and development settings. *Adv Drug Deliv Rev* 46:3–26 doi:10.1016/S0169-409X(00)00129-0

3. Redfern WS, Carlsson L, Davis a S, Lynch WG, MacKenzie I, Palethorpe S, Siegl PKS, Strang I, Sullivan AT, Wallis R, Camm AJ, Hammond TG (2003) Relationships between preclinical cardiac electrophysiology, clinical QT interval prolongation and torsade de pointes for a broad range of drugs: evidence for a provisional safety margin in drug development. *Cardiovasc Res* 58:32–45 doi:10.1016/S0008-6363(02)00846-5

4. Stöhr A, Friedrich FW, Flenner F, Geertz B, Eder A, Schaaf S, Hirt MN, Uebeler J, Schlossarek S, Carrier L, Hansen A, Eschenhagen T (2013) Contractile abnormalities and altered drug response in engineered heart tissue from Mybpc3-targeted knock-in mice. *J Mol Cell Cardiol* 63:189-198 doi:10.1016/j.yjmcc.2013.07.011

5. Vandenburgh H, Shansky J, Benesch-Lee F, Barbata V, Reid J, Thorrez L, Valentini R, Crawford G (2008) Drug-screening platform based on the contractility of tissue-engineered muscle. *Muscle & Nerve* 37: 438–447 doi: 10.1002/mus.20931

6. Zimmermann WH, Fink C, Kralisch D, Remmers U, Weil J, Eschenhagen T (2000) Three-dimensional engineered heart tissue from neonatal rat cardiac myocytes. *Biotechnol Bioeng* 68:106–114 doi:10.1002/(SICI)1097-0290(20000405)6
